# Giant abdomino scrotal hydrocele: a case report with literature review

**DOI:** 10.11604/pamj.2018.31.213.14470

**Published:** 2018-11-28

**Authors:** Abdelfattah Latabi, Mohammed Amine Lakmichi, Zakaria Dahami, Mohammed Said Moudouni, Ismail Sarf

**Affiliations:** 1 University Hospital of Marrakesh, Marrakesh, Morocco

**Keywords:** Abdominal scrotal hydrocele, urinary symptoms, scrotal approach

## Abstract

Abdomino scrotal hydrocele (ASH) is a condition in which the hydrocele sac is extended beyond the scrotum to the abdomen via the inguinal canal. The treatment is ordinarily surgical. Different approaches have been described like paramedian laparotomy, an inguinal or inguino scrotal approach. We report a case of giant unilateral hydrocele in an 18 year old male, occupying a large part of the abdomen with urinary symptoms. Ultrasonography and CT showed typical cystic mass in hourglass shape that we have approached surgically by scrotal incision and we removed all the cyst. Pathological examination found a hydrocele with no signs of malignancy. Urinary symptoms disappeared postoperatively. This is a rare entity that evolves often painless and little reported in the literature. The etiology and pathogenesis of this disease is discussed.

## Introduction

The hydrocele is a collection of fluid between the two layers of the tunica vaginalis that surrounds the testicle. The hydrocele swells the scrotum but the testicle remains normal. Rarely, scrotal hydrocele may extend into the abdominal cavity through the inguinal canal, defining the abdomino scrotal hydrocele (ASH), this condition is originally described by Dupuytren in 1834 [1]. We report a case of ASH that was special by huge dimensions, urinary symptoms and scrotal incision extended slightly to the inguinal level and was able removed the cyst completely.

## Patient and observation

**Figure 1:**
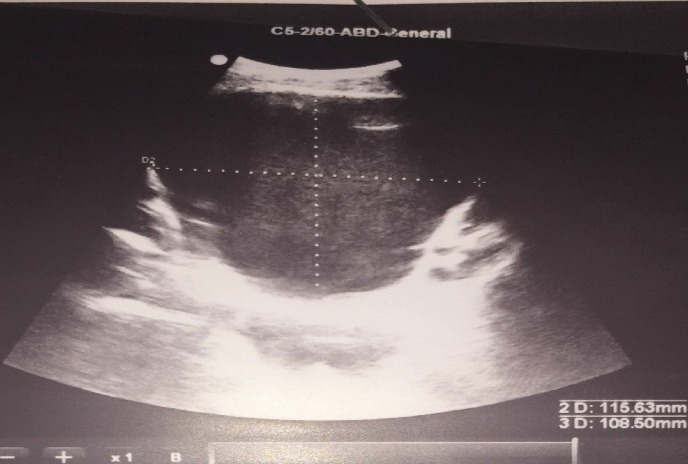
ultrasonographic imaging of the abdomen and pelvis (coronal section): huge cystic lesion

**Figure 2:**
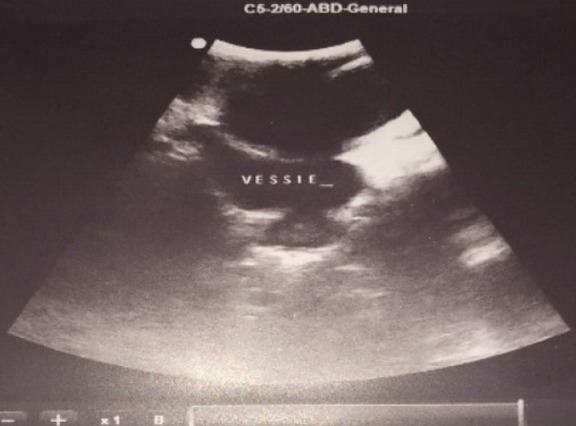
ultrasonographic imaging of the abdomen and pelvis (coronal section): intimate contact of the cyst with the bladder which is compressed

**Figure 3:**
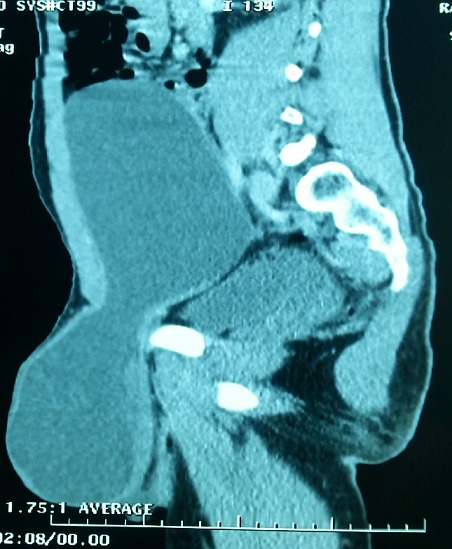
computed tomography scan imaging of the abdomen and scrotum: fluid mass in hourglass typical of the abdominoscrotal hydrocele

**Figure 4:**
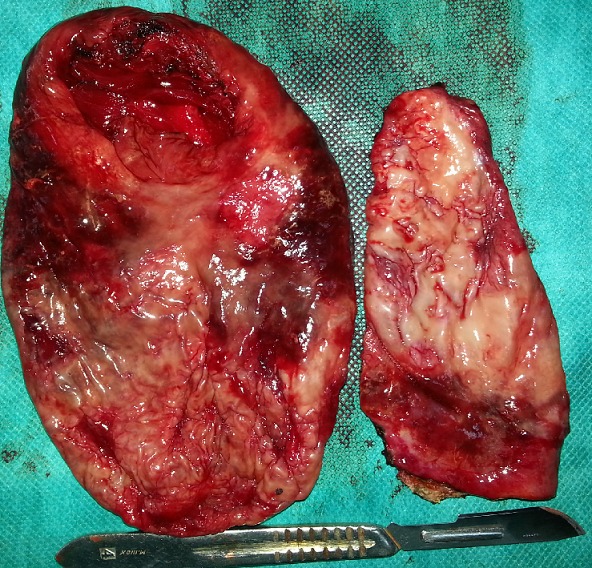
surgical specimen: abdominal and scrotal loculus

An 18 year old male was referred to our Institution with a history of an asymptomatic palpable mass in the left testis growing into the lower abdomen for 1 year. The patient complained of bladder symptoms. Physical examination revealed a healthy adolescent presented left painless scrotal mass with ipsilateral palpable abdominal mass, there was non-transilluminant. An abdomino scrotal ultrasound performed in another hospital demonstrated left fluid scrotal mass, measuring large diameter 8 centimeters (cm) above a testicle, which measuring large diameter 6 cm with a suprapubic mass hypoechogenic intra-abdominal measuring large diameter 11.5 cm. The bladder was displaced by the mass to the right (Figure 1, Figure 2). The right testis was normal except a simple hydrocele in the ipsilateral scrotal bursa with epididymal cyst measuring 6.5 millimeters (mm). Testicular tumour markers were unremarkable: alpha-fetoprotein (AFP), beta human chorionic gonadotropin (hCG), and lactate dehydrogenase (LDH). To better characterize the lesion computed tomography (CT) scan before and after contrast agent enhancement was performed. It showed a cystic mass in hourglass shape in the left scrotum, extending towards the abdomen through the inguinal canal ipsilateral. This mass displaces the ipsilateral testis to down and back. It was a mass of fluid density, thin and regular walled. It measures 23 cm in length and compressed the bladder (Figure 3). These findings were compatible with abdominal-scrotal hydrocele. The patient was scheduled for inguino scrotal approach surgery. An incision was made in the left scrotal extended slightly on groin. A large bilocular hydrocele was found. The smaller loculus in the scrotum and the larger in the abdomen, both linked through the inguinal canal. It contained more than one liter of clear straw coloured fluid. The surrounding peritoneal layer was dissected from the cyst and complete excision of the abdominal and scrotal sac was performed (Figure 4). The post-operative course was uneventful. Histological examination of the specimen confirmed the diagnosis of hydrocele with no signs of malignancy, and after surgery we noted a disappearance of urinary symptoms.

## Discussion

We found more than 100 cases described in the literature. Unilateral ASH was found in 59% of cases with almost even distribution between left and right-sided hydroceles. Ipsilateral undescended testicle, testicular dysmorphism, contralateral pathology (inguinal hernia) and adjacent organs compression often accompanied ASH [2]. Dharamveer Singh *et al.* have described a case of HSA which compresses the right urinary tract, this one has been solved after surgery [3]. 11-month-old patient with a right ASH and undescended testis are reported by Kara T1 [4]. The etiology of ASH is unknown; however, different theories have been described in literature to explain the pathogenesis such as, valve theory [3, 5], diverticulum theory [5], displacement as per Laplace's law [6] and increased production or decreased resorption of fluid [7]. However, the most accepted theory is the one by Dupuytren which suggests that excessive intracystic pressure causes cephalad extension of scrotal swelling through the deep inguinal ring [5]. Brodman described it to be because of high obliteration of processes vaginalis above the deep inguinal ring, leaving a high infantile hydrocele [5]. Diagnosis of ASH is done by clinical examination aided by imaging techniques. Positive transillumination test and cross-fluctuation between the abdominal and the scrotal collections are the clinical hallmarks of ASH diagnosis. Ultrasound is the first choice to demonstrate the communication between two components however in selected cases ultrasonography may be inadequate and in these contrast enhanced computed tomography or magnetic resonance imaging could be used to demonstrate extension of the hydrocele through the inguinal canal into the abdominal cavity [3, 6]. In our case, a contrast enhanced computed tomography gave us a complete diagnosis. Axial MRI along with MR angiography is useful in detecting vascular complications like deep vein thrombosis [8]. The differential diagnoses include spermatic cord lymphangioma, giant hydronephrosis extending into the true pelvis, bladder diverticulum and pelvic neuroblastoma [3]. Sometimes ASH may be confused with large, complete, indirect inguinal hernia [6]. Ordinarily, the reported management is by surgical excision. Khorasani M *et al.* described in their series including 29 cases of ASH, twenty out of 29 patients (70%) were initially managed expectantly. Sixteen (80%) had resolution of their abdominal component, twelve (60%) of which went on to have full resolution, so they recommend observation as the first step in the management of uncomplicated ASH [9]. Different approaches have been described like paramedian laparotomy, an inguinal or inguinoscrotal approach [5]. In difficult cases preperitoneal approach had been described which facilitates complete removal of abdominal component. In our case, we have also followed the preperitoneal approach by enlargement of the scrotal incision to the inguinal area to excise both the component in total.

## Conclusion

ASH is a very rare form of hydrocele. The diagnosis is evoked by clinical examination in front of a scrotal hydrocele associated with an ipsilateral abdominal cystic mass. Ultrasound and CT scan show an inguino-scrotal hourglass mass. The abdominal compartment of the ASH can cause loco-regional complications by compression. MRI can be a valuable contribution to surgical curative treatment.

## Competing interests

The authors declare no conflict of interest.
